# Recovery of Verbal Working Memory Depends on Left Hemisphere White Matter Tracts

**DOI:** 10.1162/NOL.a.20

**Published:** 2025-09-26

**Authors:** Randi C. Martin, Junhua Ding, Ali I. Alwani, Steve H. Fung, Tatiana T. Schnur

**Affiliations:** Department of Psychological Sciences, Rice University, Houston, TX, USA; Department of Psychology, University of Edinburgh, Edinburgh, UK; Department of Radiology, Houston Methodist Research Institute, Houston, TX, USA; Department of Physical Medicine and Rehabilitation, University of Texas Health Sciences Center, Houston, TX, USA

**Keywords:** left-hemisphere acute stroke, longitudinal recovery, phonological working memory, right hemisphere recruitment, semantic working memory, white matter tractography

## Abstract

Researchers propose that the recovery of language function following stroke depends on the recruitment of perilesional regions in the left hemisphere and/or homologous regions in the right hemisphere. Many investigations of recovery focus on changes in gray matter regions, whereas relatively few examine white matter tracts and none address the role of these tracts in the recovery of verbal working memory. The present study addressed these gaps, examining the role of left versus right hemisphere tracts in the longitudinal recovery of phonological and semantic working memory. For 24 individuals with left hemisphere stroke, we assessed working memory performance within 1 week of stroke (acute time point) and at more than 6 months after stroke (chronic time point). To address whether recovery depends on the recruitment of left or right hemisphere tracts, we assessed whether changes in working memory were related to the integrity of five white matter tracts in the left hemisphere which had been implicated previously in verbal working memory and their right hemisphere analogues. Behavioral results showed significant improvement in semantic but not phonological working memory from the acute to chronic time points. Improvements in semantic working memory significantly correlated with tract integrity as measured by functional anisotropy in the left direct segment of the arcuate fasciculus, inferior fronto-occipital fasciculus and inferior longitudinal fasciculus. The results confirm the role of white matter tracts in language recovery and support the involvement of the left rather than right hemisphere in the recovery of semantic working memory.

## INTRODUCTION

The neural basis of the recovery of language function following brain damage is of theoretical and clinical significance. Studies on the topic have typically focused on the role of gray matter regions (Hartwigsen & Saur, 2019; Kiran, 2012), with findings indicating that perilesional left hemisphere gray matter regions and/or homologous regions in the right hemisphere support language recovery (Crinion & Price, 2005; Hartwigsen & Saur, 2019; Stefaniak et al., 2022; Turkeltaub et al., 2011; cf. Wilson & Schneck, 2021). Given the importance of the interaction among gray matter regions in cognition and language processing (Kiran & Thompson, 2019; Ribeiro et al., 2024), one would also expect that the integrity of white matter tracts connecting gray matter regions would influence recovery (Han et al., 2024; Siegel et al., 2022; Tilton-Bolowsky et al., 2024). A smaller body of research on the role of white matter tracts has again provided evidence supporting the involvement of the left and/or right hemispheres, with the integrity of tracts in one or both hemispheres relating to recovery for both spontaneous and treatment-driven language recovery (e.g., Breier et al., 2011; Schlaug et al., 2009; Sihvonen et al., 2023). These studies have most often examined the relation of white matter tracts to recovery of specific language processes such as word production (Hillis et al., 2018; McKinnon et al., 2018), sentence production (Ding & Schnur, 2022), or sentence comprehension (Xing et al., 2017). The present research addressed a novel topic—specifically, examining the role of white matter tracts in the recovery of verbal working memory (WM), which has been shown to play an important role in language comprehension and production (Martin, 2021; Martin & Schnur, 2019). In the current study, WM was assessed within the domain-specific model, which postulates separate phonological and semantic components, which play different roles in language processing (Martin, 2021; Martin et al., 2020). Thus, the study assessed whether different white matter tracts supported the recovery of the phonological and semantic components of WM.

### Domain-Specific WM

Working memory is a system that allows for the maintenance and manipulation of information over short time periods in the service of carrying out complex cognitive tasks, such as reasoning and language processing (Cowan, 2011). Considerable evidence supports the claim that there are specialized WM capacities for different domains—for instance, with separate capacities for maintaining verbal and visuo-spatial information (Baddeley et al., 2020) and, within the verbal domain, separate capacities for maintaining phonological and semantic information (Martin et al., 2020). The current study focuses on this latter distinction between phonological and semantic information. In the domain-specific model proposed by Martin et al. (1999), there is a tight linkage between word processing and these phonological and semantic working memory capacities. That is, as a series of words are presented, their lexical-semantic and phonological representations are activated and stored in separate buffers, which can be independently disrupted by brain damage. Prior results implicate different roles for the two buffers in language comprehension and production, and thus recovery of each capacity could lead to different language outcomes (Martin & Schnur, 2019; Horne et al., 2022; Zahn & Martin, 2024).

The initial evidence supporting the distinction between phonological and semantic WM came from case studies of brain damaged individuals who showed different patterns on tasks tapping the maintenance of phonological or semantic information for word or nonword lists. Individuals argued to have phonological WM deficits did not show standard phonological effects on WM, such as phonological similarity or word length effects, suggesting that they could not rely on phonological information for recall (Martin & He, 2004; Vallar & Baddeley, 1984). In contrast, they did show the standard pattern of better performance on word than nonword lists, which could be attributed to their ability to retain semantic information that was available in words but not nonwords (Martin & He, 2004; Martin et al., 1994). Those argued to have semantic WM deficits did not show the standard advantage for memory of words over nonwords though they did show typical phonological effects on WM. Evidence from recognition probe tasks designed to tap the two capacities also supported the distinction in the nature of their WM deficits (see also Allen et al., 2012). The category probe task, emphasizing semantic WM, required participants to listen to word lists and judge whether a probe word was in the same semantic category (e.g., list: tulip, desk, hat; probe: rose). The rhyme probe task, emphasizing phonological WM, was similar, but required participants to judge whether a probe word rhymed with any list word (e.g., list: bread table fork; probe: sled). Those with semantic WM deficits performed better on the rhyme than category probe task whereas those with phonological WM deficits showed the reverse (e.g., Martin et al., 1994). In terms of language processing, those with semantic WM deficits performed more poorly than those with phonological WM deficit on comprehension requiring integrating elements across some distance, whereas those with phonological WM deficits performed more poorly than those with semantic WM deficits on verbatim sentence repetition (Martin & Romani, 1994; Martin et al., 1994).

More recent work taking large sample case series approaches with brain damaged individuals or individual differences approaches with healthy younger and older participants has provided further evidence of separate phonological and semantic WM factors (Zahn, 2024; Zahn & Martin, 2024) and separate influences of phonological and semantic WM in language production (Martin & Schnur, 2019; Zahn et al., 2022) and comprehension (Horne et al., 2022; Lu et al., 2024; Tan & Martin, 2018; Tan et al., 2017). Regarding the separability of semantic and phonological WM, Zahn et al. (2024) took a factor analytic approach with 152 healthy participants using four measures of phonological WM (digit span, digit matching span, rhyme probe and nonword repetition) and three measures of semantic WM (category probe, conceptual span, and synonym probe). They confirmed the existence of two factors, with all phonological measures loading significantly on one factor and all semantic measures on a second factor. Regarding the relation of semantic and phonological WM to language processing, there is evidence of a greater contribution of semantic WM in both comprehension and production (see Martin, 2021, for review; Zahn et al., 2022).

### Neural Correlates of Domain-Specific WM

#### Relations to gray matter regions

In line with distinctive behavioral patterns, different gray matter regions have been identified as supporting phonological and semantic WM. Early case study findings indicated maximum lesion overlap in the left supramarginal gyrus (SMG) for those individuals showing a phonological WM deficit (see Vallar & Papagno, 2002). The role of the SMG in phonological WM has been corroborated by recent findings using multivariate lesion symptom mapping for brain damaged participants (Martin et al., 2021; Pisoni et al., 2019) and multivariate decoding methods in functional magnetic resonance imaging (fMRI) studies of healthy individuals (Yue & Martin, 2021; Yue et al., 2019). It should be noted that the SMG is thought to be involved in the storage of phonological representations, whereas frontal regions involved in motor planning and execution have been implicated in articulatory rehearsal of these representations (Martin et al., 2021; Vallar & Papagno, 2002; Yue et al., 2019). With respect to semantic WM, fewer studies have been carried out; however, evidence from lesion symptom mapping (Martin et al., 2021) and univariate and multivariate fMRI methods in unimpaired populations (Crosson et al., 1999; Hamilton et al., 2009; Shivde & Thompson-Schill, 2004; Yue & Martin, 2021) implicates the left inferior frontal gyrus (IFG) and the angular gyrus (AG), regions which are distinct from those involved in phonological WM.

#### Relations to white matter tract integrity

With respect to the white matter correlates of verbal WM, research typically relates white matter integrity to performance on tasks such as digit, word, and nonword span (e.g., Charlton et al., 2013; Østby et al., 2011). These tasks are primarily phonological in nature, as there is little semantic information in random word and nonword lists and phonological features of the stimulus lists influence performance (e.g., phonological similarity and word length; Mueller et al., 2003). Thus, these measures are often assumed to measure the short-term retention of phonological information. Work on the white matter correlates of phonological WM typically implicates fronto-parietal tracts including the superior longitudinal fasciculus, and more specifically, the arcuate fasciculus (AF). These findings have been replicated across many different study populations, including healthy younger and older adults (Burzynska et al., 2010; Charlton et al., 2013; Takeuchi et al., 2011), children (Østby et al., 2011), and people with neurological disorders (Audoin et al., 2007; Meyer et al., 2015; Sepulcre et al., 2009). In studies with healthy individuals, relations between verbal WM performance and white matter integrity have been documented for both left and right hemisphere tracts (Burzynska et al., 2010; Takeuchi et al., 2011). However, these studies often involved complex WM functions through the comparison of performance on three-back versus one-back tasks (Burzynska et al., 2010) or performance on a combination of forward and backward span (Charlton et al., 2013; Østby et al., 2011; Takeuchi et al., 2011). It is possible that the involvement of the right hemisphere tracts resulted from the role of attentional and other executive processes in these WM tasks, rather than the storage of phonological and semantic information, which has been the focus of relations to language processing (Martin et al., 2021).

Recently, Horne et al. (2023, 2024) examined the white matter correlates of phonological and semantic WM for 45 individuals at 1 year or more post left hemisphere stroke. Performance on a semantic WM task (category probe) and phonological WM task (digit matching) was related to fractional anisotropy (FA) values for five left hemisphere white matter tracts. The tracts were selected on the grounds of prior literature implicating that tract in supporting verbal WM or because they had terminations in gray matter regions involved in phonological or semantic WM. Thus, the AF and its anterior subsegment (AAF; superior longitudinal system 3 according to Mandonnet et al., 2018) and posterior subsegment (PAF; posterior transverse system 1 according to Mandonnet et al., 2018) were included because the AAF connects the SMG—the proposed location of the phonological WM buffer (Crosson et al., 1999; Hamilton et al., 2009; Shivde & Thompson-Schill, 2004; Yue & Martin, 2021)—to regions in the frontal lobe that support articulatory rehearsal (Chein & Fiez, 2001; Yue, 2018) and executive function (Smith et al., 1998), and the PAF connects the SMG to temporal lobe regions that support speech perception (Turkeltaub & Coslett, 2010). Tracts with terminations in either the IFG or the AG were included because of the evidence for the involvement of these regions in semantic WM. These included the direct segment of the AF (DAF; i.e., superior longitudinal system 4), inferior longitudinal fasciculus (ILF; i.e., basal longitudinal system), inferior frontal-occipital fasciculus (IFOF; i.e., inferior longitudinal system 1–3), uncinate fasciculus (i.e., inferior longitudinal system 4), and middle longitudinal fasciculus (i.e., middle longitudinal system). Overall, the tracts included the AF and its subsegments, which had been the focus of prior study on the relation of white matter integrity to verbal WM, as well as most of the other tracts that have been implicated in language processing (Shekari & Nozari, 2023). Multiple regression analyses were carried out that used tract integrity as the dependent measure and semantic or phonological WM as the main predictor while controlling for the other WM measure, single word comprehension, and gray matter damage at tract termini. These analyses revealed a significant independent relation of phonological WM to the integrity of the AF (overall) and the ILF. A significant independent relation to semantic WM was obtained for the PAF and the IFOF. The findings conformed to some degree to predictions, with phonological WM related to the integrity of the AF as a whole, consistent with prior findings, and semantic WM related to the integrity of the IFOF. It was suggested that the unexpected relations of the integrity of the ILF to phonological WM and the PAF to semantic WM might arise due to possible variation in terminations across individuals, with some having terminations of the ILF in the SMG and not the AG and vice versa for the PAF, given the proximity of the two regions. Thus, these findings documented the importance of left hemisphere tracts in predicting WM performance at a chronic stage post stroke, with different tracts implicated as more important for either semantic or phonological WM. Relations to right hemisphere tract integrity were not reported, and thus this study did not address whether tracts in both hemispheres potentially contributed to performance.

### Current Study

Whereas the Horne et al. (2023, 2024) studies examined relations of verbal WM to white matter tract integrity at one time point (the chronic stage of stroke) solely in the left hemisphere, the present study examined the relation of tract integrity to longitudinal recovery as measured in changes in WM performance from an acute to a chronic time point within the same individuals, considering both left and right hemisphere tracts. Thus, the individuals tested in the present study were assessed on WM measures at an acute stage post stroke (mean = 4 days, range: 2–10 days) and again at a chronic time point of at least 6 months post stroke. To the extent that recovery depends solely on connections among left hemisphere brain regions, one would predict that the degree of improvement would be related only to the integrity of white matter tracts in the left hemisphere. On the other hand, if recovery depends on the functioning of homologous right hemisphere regions, one would predict that improvement would also relate to the integrity of white matter tracts in the right hemisphere—either alone or in conjunction with left hemisphere tracts. The participants in the current study were part of larger study recruiting individuals at the acute stage of left hemisphere stroke (Ding et al., 2020), which excluded those with prior strokes. Thus, these individuals did not have lesions in the right hemisphere.

We selected and identified bilaterally two tracts for which significant relations of FA values to either phonological or semantic WM were obtained (i.e., the ILF and IFOF; Horne et al., 2023, 2024). In addition, we examined three subsegments of the AF including the AAF, DAF, and PAF following their hypothesized different contributions to language processing (Forkel et al., 2020). Thus, five tracts were examined in both the left and right hemispheres. With respect to predictions, there is, to our knowledge, no prior literature regarding white matter involvement in verbal WM recovery. One might hypothesize that those tracts uncovered in the Horne et al. study to support phonological or semantic WM would be those that would support recovery of their respective capacities, though perhaps with the involvement of right hemisphere homologues.

However, several considerations might argue against these predictions. For one, if the left hemisphere tracts typically involved are very damaged, recovery using these tracts may be impossible and other left hemisphere tracts may become more involved (rather than just right hemisphere homologues; Keser, Sebastian, et al., 2020; van Hees et al., 2014). Also, it is possible that if tracts supporting, for instance, semantic WM are damaged, preserved phonological WM might be used as a backup to aid access to semantic information until gray matter or white matter changes take place to support maintenance of semantic information. Finally, although about half of the participants in the Horne et al. (2023, 2024) studies were recruited at the acute stage of stroke (but tested at least 1 year post stroke), the other half were those recruited at the chronic stage of at least 1 year post stroke and many of them were several years post stroke. The two subsets differed substantially in lesion size, with much larger lesions for those recruited at the chronic stage (see Zahn et al., 2022, for discussion). Thus, the inclusion of those with much larger lesions in the Horne et al. study may have led to a different pattern of relations of tract integrity to WM than might be observed for those with smaller lesions, like the individuals tested here. Given these considerations, it is difficult to make predictions about which specific tracts might be involved in phonological or semantic WM recovery. Because of the exploratory nature of the involvement of specific tracts, it was critical that corrections for multiple comparisons be made, which was done here using the false discovery rate (FDR) method. The results provide the first direct evidence regarding whether there is involvement of left or right hemisphere tracts, or both, in verbal WM recovery.

## MATERIALS AND METHODS

### Participants

**Figure 1.**
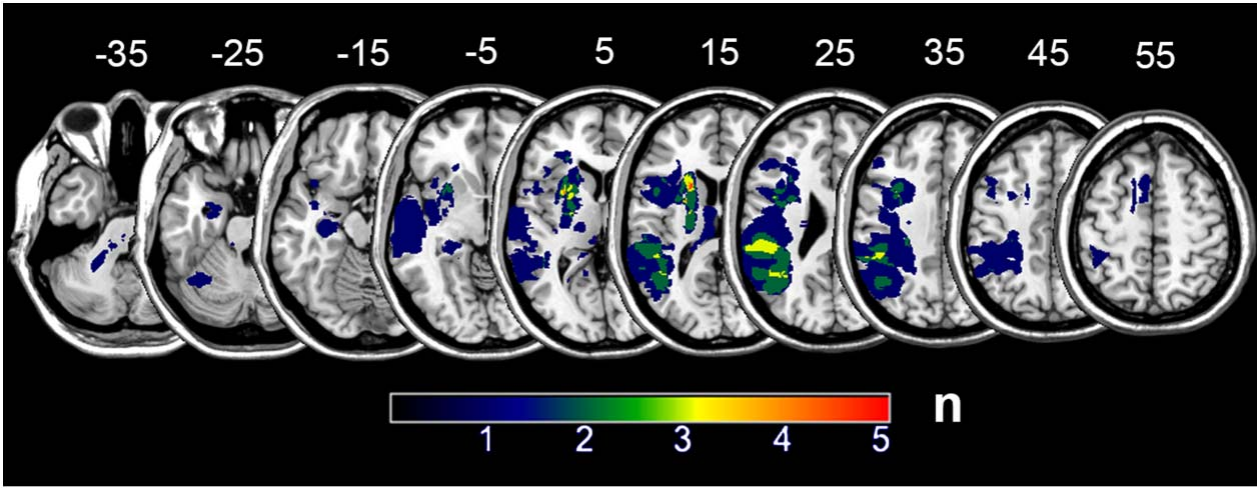
Lesion overlap. Number of individuals with lesions at each voxel.

As part of a larger project involving multiple comprehensive stroke centers in Houston, Texas, we consecutively recruited monolingual English speakers with left hemisphere stroke independent of clinical diagnosis of aphasia and without other health conditions which impacted cognition (i.e., tumor, dementia, alcohol and/or drug dependency). Among enrolled participants, 24 (acute age = 58 yr, range = 20–78 years; education = 15 yr, range = 12–23 yr; 11 males; 20 right-handed) completed working memory assessments at both acute (within an average of 4 days post stroke, range: 2–12 days) and chronic (>6 months since stroke; with an average of 324 days, range: 175–480 days) time points as well as follow-up neuroimaging at the chronic time point. Left hemisphere lesions affected frontal, temporal, and parietal lobes, subcortical nuclei, white matter, and cerebellum (lesion size: average 6,728 mm^3^, range 51–37,085 mm^3^; Figure 1). In addition, 13 control participants were recruited (age = 55 yr, range = 37–78 years; education = 16 yr, range = 12–22 yr; 3 males; 18 right-handed). The control and patient cohorts did not differ on these demographic variables (*p*s > 0.31). Informed consent was obtained from either the subject or a legally authorized representative of the subject as approved by the institutional review boards of the Baylor College of Medicine, Rice University, the University of Texas Health Science Center, and the Houston Methodist Hospital.

### Behavioral Testing

#### Working memory

Digit matching span measured phonological working memory ability (Allen et al., 2012) for 22 of the 24 participants. Two participants completed the digit span task using the standard Wechsler Adult Intelligence Scale–Revised (WAIS-R; Wechsler, 1981) procedure. Strong correlations between digit matching span and digit span have been obtained for healthy older adults (*r*(57) = 0.63, *p* < 0.001; Zahn et al., 2024) and those with left hemisphere brain damage (*r*(36) = 0.72, *p* < 0.001; Zahn & Martin, 2024). With respect to digit matching span, participants heard 2-digit lists (1 digit/s) and then judged whether the two lists were the same or not. Half of the trials matched, half did not. In the non-match trials, the second list reversed two adjacent digits and the reversed position was randomized. The list length gradually increased from 2 to 6 digits, with 6, 8, 6, 8, and 10 trials per list length.

A category probe task tested semantic working memory ability (Martin et al., 1994, 2021). In this task, participants heard a word list (1 word/s) and then a probe word. Participants judged whether the probe belonged to the same semantic category as any list item (e.g., matching list: tulip, desk, hat; probe: rose). Categories included animals, body parts, clothing, fruit, and kitchen equipment. On half of the trials the probe matched the category of a list item and on half it did not. List length gradually increased from 1 to 4 items, with 8, 8, 12, and 16 trials per list length.

For both tests of working memory, we stopped testing if accuracy fell below 75% for a particular list length. We used linear interpolation to estimate the list length corresponding to 75% accuracy.

Different list lengths were used for the two tasks given their differing requirements. Specifically, for category probe, one-item lists can be presented (e.g., list item: rose, probe: daisy), whereas for digit matching, the shortest lists must have two items so that an order switch can be made for non-matching trials (e.g., 3 5 – 5 3). It was expected that spans would be larger for the digit matching span task than the category probe task, given prior results. For 13 controls tested by Martin and Schnur (2019) with the same materials and procedures used here, the mean span was 6.1 for digit matching and 4.7 for category probe. All the WM scores were standardized based on this control cohort.

#### Single word processing

A single word single picture matching task was used to measure word processing ability (cf. Martin & Schnur, 2019). Seventeen pictures were presented four times with different types of auditory words, including matching words, phonologically or semantically related foils and unrelated foils. Participants were asked to judge whether the picture and word represented the same object. Phonological/semantic *d*′ scores were calculated to the ability to discriminate between matching trials and phonological/semantic foils (*d*′ phonological and *d*′ semantic, respectively). All the *d*′ scores were standardized based on the control cohort.

### Neuroimaging Data

Due to an extended participant recruitment period, two different sets of high-resolution T1 and diffusion tensor imagine (DTI) sequences were employed at the chronic stage. Eight participants were scanned in a Philips Intera 3T scanner. The acquisition parameters were as follow: (1) DTI: TR = 11,098 ms, TE = 60 ms, 70 axial slices, slice thickness = 2 mm, in-plane resolution: 2 mm * 2 mm, 32 directions plus 1 b0 volume, b-value = 800 s/mm^2^; (2) 3D T1 TFE: TR = 8.4 ms, TE = 3.9 ms, flip angle = 8°, 175 sagittal slices, slice thickness = 1 mm, in-plane resolution: 0.94 mm * 0.94 mm. Other participants were scanned in a Siemens Prisma 3T scanner. The acquisition parameters were as follow: (1) DTI: TR = 7,700 ms, TE = 70 ms, 72 axial slices, slice thickness = 2 mm, in-plane resolution: 2 mm * 2 mm, 64 directions plus 1 b0 volume, b-value = 1,000 s/mm^2^; (2) 3D T1 MPRAGE: TR = 2,600 ms, TE = 3.02 ms, flip angle = 8°, 176 sagittal slices, slice thickness = 1 mm, in-plane resolution: 1 mm * 1 mm. We used PANDA (https://www.nitrc.org/projects/panda/; Cui et al., 2013) to perform preprocessing of DTI. Specifically, we corrected for the eddy-current distortions, fitted the tensor model, calculated FA values, and performed deterministic tractography using the FACT algorithm, with 45° angle and 0.2 FA thresholds (Mori & van Zijl, 2002; Wang et al., 2007).

**Figure 2.**
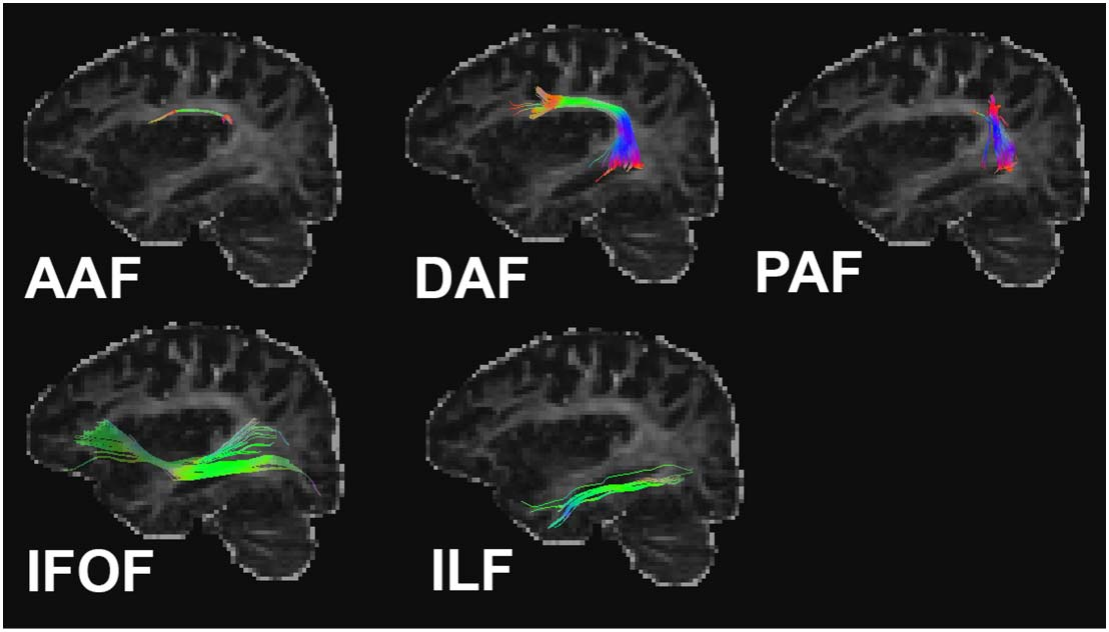
Representative tracts of interest from a single participant after left hemisphere stroke. AAF/DAF/PAF = anterior/direct/posterior segment of arcuate fasciculus; IFOF = inferior fronto-occipital fasciculus; ILF = inferior longitudinal fasciculus.

Five working memory-related tracts were selected and dissected bilaterally including the AAF, DAF, PAF, ILF, and IFOF (Horne et al., 2023; Figure 2). We used a two regions-of-interest (ROIs) method to isolate different tracts in native space using TrackVis (https://trackvis.org/).

The AAF was defined by two disc ROIs placed subjacent to IFG and SMG. The DAF was defined by two disc ROIs placed subjacent to IFG and posterior middle temporal gyrus (MTG). The PAF was defined by two disc ROIs placed subjacent to the AG and posterior MTG (Catani et al., 2007). The IFOF was defined by two disc ROIs placed in the ventral medial occipital lobe and anterior floor of the external/extreme capsule (Catani & Thiebaut de Schotten, 2008; Fekonja et al., 2019). The ILF was defined by two disc ROIs placed in the anterior temporal lobe and the occipital lobe (Catani & Thiebaut de Schotten, 2008; Fekonja et al., 2019). When the tract was identifiable within a participant, we extracted the FA value (Table 1).

**Table 1.** Tract fractional anisotropy values

	Mean	*SD*	Min	Max
Left hemisphere				
AAF (*n* = 20)	0.39	0.03	0.33	0.45
DAF (*n* = 17)	0.43	0.03	0.37	0.47
PAF (*n* = 18)	0.38	0.03	0.33	0.42
IFOF (*n* = 24)	0.43	0.03	0.37	0.47
ILF (*n* = 24)	0.41	0.03	0.36	0.48
Right hemisphere				
AAF (*n* = 23)	0.40	0.03	0.33	0.45
DAF (*n* = 9)	0.42	0.03	0.36	0.46
PAF (*n* = 21)	0.39	0.04	0.29	0.45
IFOF (*n* = 24)	0.43	0.03	0.38	0.47
ILF (*n* = 24)	0.41	0.03	0.34	0.48

*Note*. AAF/DAF/PAF = anterior/direct/posterior segment of arcuate fasciculus; IFOF = inferior fronto-occipital fasciculus; ILF = inferior longitudinal fasciculus.

To rule out the potential influence of gray matter damage, we calculated the gray matter damage at the tracts’ termini and used that as a control variable in our analyses. First, lesion masks were manually drawn on T1 images using ITK-snap (Yushkevich et al., 2006). Then we registered T1 images to the Colin-27 template using Advanced Normalization Tools (ANTs) and normalized masks to the Montreal Neurological Institute (MNI) space based on the transformation parameters (Avants et al., 2008; cf. Ding & Schnur, 2022, for a similar approach). Using the Automated Anatomical Labeling (AAL) atlas (Tzourio-Mazoyer et al., 2002) to define regions, we estimated a region’s gray matter damage as the region’s intersection with the lesion masks. The AAF’s termini included the opercular part of IFG and SMG. The DAF’s termini included the opercular part of IFG and MTG. The PAF’s termini included the AG and MTG. The IFOF’s termini included the frontal orbital gyrus and occipital lobe. The ILF’s termini included the temporal pole and occipital lobe. We calculated total gray matter damage related to each tract as the summation of the number of voxels for the two termini damaged.

### Statistical Analysis

To explore the relation between white matter tracts and working memory recovery, we conducted partial correlations between FA values of the white matter tracts and changes in WM performance from the acute to chronic time points. For both left and right hemisphere analyses, we controlled for acute WM baseline and changes of word picture matching scores (i.e., changes in *d*′ semantic and *d*′ phonological scores). In addition, for the left hemisphere, we controlled for gray matter damage of the tracts’ two termini as a result of the left hemisphere stroke. As tracts were not identified in all participants, we only analyzed tracts identified in more than half of the sample. Therefore, the right DAF was excluded from this analysis (*n* = 9). FDR correction was conducted within each hemisphere and within each WM task to account for multiple comparisons.

## RESULTS

### Behavioral Results

**Figure 3.**
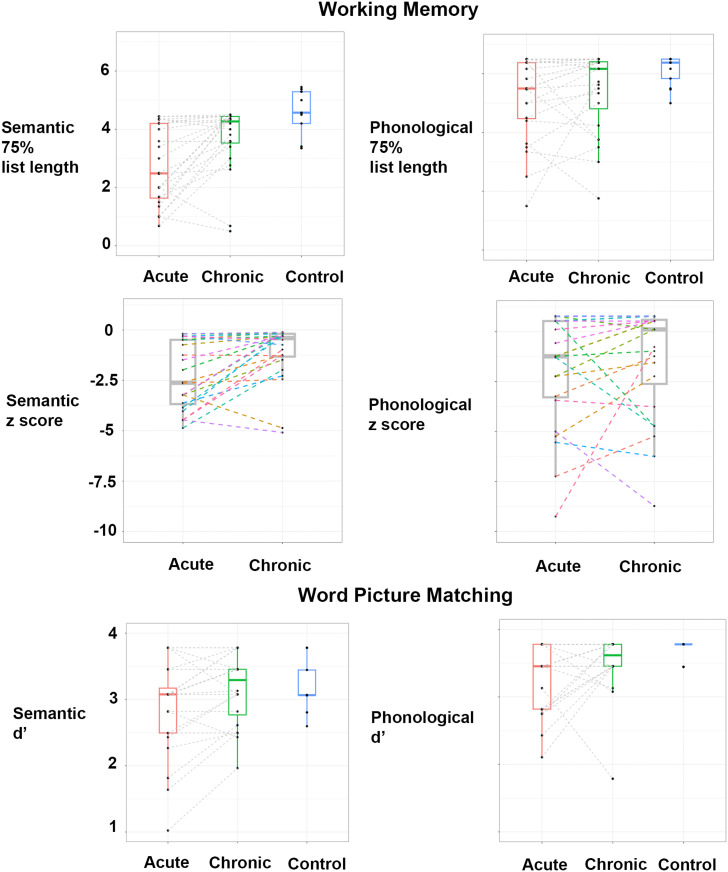
Individuals’ semantic and phonological working memory linearly interpolated estimates of list length corresponding to 75% accuracy and word picture matching performance at acute and chronic post-stroke time points.

Patients’ working memory and word picture matching performance at the acute and chronic stages are shown in Figure 3 and Table 2. Participants’ semantic and phonological working memory scores were significantly lower than controls (*t*s > 2.2, *p*s < 0.04). In contrast, their semantic and phonological single word processing scores were not significantly different than controls (|*t*| < 1.9, *p*s > 0.07), except for the acute *d*′ phonological (*t* = 3.4, *p* = 0.002). There was no significant difference between acute semantic and phonological WM *z*-scores (*t*(23) = 1.68, *p* = 0.11). Paired *t* tests indicated a significant improvement in semantic WM (*t*(23) = 3.7, *p* = 0.001) from acute to chronic stroke, but not in phonological WM (*t*(23) = 0.8, *p* = 0.43).

**Table 2.** Individuals’ semantic and phonological working memory and word picture matching performance at acute and chronic post-stroke time points

	Semantic		Phonological	
	Raw	*z*-score	Raw	*z*-score
Working memory				
Acute	2.7 ± 1.3***	−2.3 ± 1.7	5.2 ± 1.4**	−1.8 ± 2.9
Chronic	3.7 ± 1.1**	−1.1 ± 1.4	5.4 ± 1.4*	−1.4 ± 2.7
Change	1.0 ± 1.3	1.2 ± 1.6	0.2 ± 1.3	0.4 ± 2.6
Control	4.6 ± 0.8		6.1 ± 0.5	
Word picture matching (*d*′)				
Acute	2.9 ± 0.7	−0.7 ± 1.8	3.3 ± 0.5**	−2.6 ± 3.4
Chronic	3.2 ± 0.5	−0.1 ± 1.3	3.5 ± 0.4	−1.3 ± 2.9
Change	0.3 ± 0.4	0.7 ± 1.1	0.2 ± 0.6	1.3 ± 4.0
Control	3.2 ± 0.4		3.7 ± 0.1	

*Note*. Indicates significant difference from controls: *p < 0.05, **p < 0.01, ***p < 0.001.

### Relationship Between FA and Working Memory Recovery

**Figure 4.**
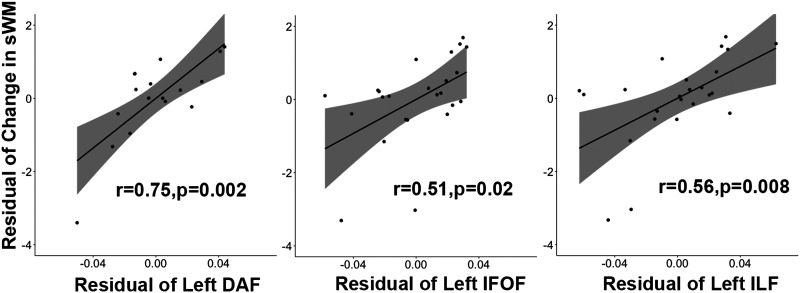
The significant effect of tracts’ FA on semantic working memory performance. sWM = semantic working memory; DAF = direct segment of arcuate fasciculus; IFOF = inferior fronto-occipital fasciculus; ILF = inferior longitudinal fasciculus.

After controlling for acute performance of working memory, recovery of word-picture matching, and gray matter left hemisphere damage of tracts’ termini, three white matter tracts’ FA significantly related with the degree of semantic WM change from acute to chronic time points (FDR corrected; Table 3 and Figure 4): left DAF (*r* = 0.75; *p* = 0.002), left IFOF (*r* = 0.51, *p* = 0.02), and left ILF (*r* = 0.56, *p* = 0.006; no significant effects after FDR correction in the right hemisphere). Higher tract FA always led to better semantic WM recovery. No significant effects were found for phonological WM after FDR correction.

**Table 3.** Correlations between FA and WM change controlling for acute WM, word-picture matching, and tract termini damage (LH)

	Semantic WM		Phonological WM	
	*R*	*p*	*R*	*p*
LH				
AAF (*n* = 20)	0.46	0.06	0.28	0.28
DAF (*n* = 17)	0.75	**0.002**	0.15	0.60
PAF (*n* = 18)	0.26	0.34	0.52	0.05
IFOF (*n* = 24)	0.51	**0.02**	0.37	0.10
ILF (*n* = 24)	0.56	**0.008**	0.43	0.05
RH				
AAF (*n* = 23)	0.46	0.04	0.40	0.07
PAF (*n* = 21)	0.27	0.27	0.35	0.14
IFOF (*n* = 24)	0.43	0.04	0.26	0.25
ILF (*n* = 24)	0.44	0.04	0.32	0.15

*Note*. **Bold** numbers are significant after FDR correction. WM = working memory; LH/RH = left/right hemisphere; AAF/DAF/PAF = anterior/direct/posterior segment of arcuate fasciculus; IFOF = inferior fronto-occipital fasciculus; ILF = inferior longitudinal fasciculus.

Unexpectedly, three patients demonstrated a large decrease in phonological WM between acute and chronic time points. After excluding these patients from the analysis, the partial correlations of phonological WM with fiber tract integrity did not increase but instead decreased (range of *r*: −0.48 – −0.01). Thus, the inclusion of these three participants in the analyses is not the source of a lack of positive relation of tract FA to phonological WM. It should also be noted that these three patients did not show dramatic declines on semantic WM or the semantic and phonological single word processing measures. Instead, they showed no change or an improvement, except for one patient who showed a small decline in semantic WM *z*-score (−0.66)—much smaller than the large decline in phonological WM *z*-score (−3.65). Thus, the large decreases in phonological WM for these three participants did not appear to be due to some general event, such as a second stroke, that had widespread effects.

## DISCUSSION

While considerable research has documented changes in gray matter function in both the left and the right hemispheres during recovery of language processing following stroke (e.g., Crinion & Price, 2005; Hartwigsen & Saur, 2019; Stefaniak et al., 2022; Turkeltaub et al., 2011; cf. Wilson & Schneck, 2021), a much smaller body of research has examined the role of white matter tracts in recovery, again with some evidence indicating the involvement of both left and right hemisphere tracts (e.g., Breier et al., 2011; Schlaug et al., 2009; Sihvonen et al., 2023). Our principal aim in the current study was to determine whether both left and right hemisphere white matter tracts contribute to the recovery of phonological and semantic WM, an issue not previously addressed. For our participants with left hemisphere stroke tested at acute and chronic time points, semantic WM showed significant improvement, whereas phonological WM did not. Recovery of semantic WM was related to the integrity of left hemisphere tracts and not to right hemisphere tracts. For phonological WM, none of the relations to white matter tracts in either the left or right hemisphere met statistical significance. That semantic WM recovery relies exclusively on the integrity of white matter tracts in the left hemisphere contributes to current debates concerning the degree to which recruitment of the right hemisphere contributes to the recovery of function in language processes (Makin & Krakauer, 2023; Martin et al., 2022; Wilson et al., 2023), extending the findings to verbal WM, where a tight linkage between word processing and WM capacities is assumed in the domain-specific model (Martin et al., 2021).

It should be noted that the lack of relation of phonological WM to white matter tract integrity could not be attributed to a restriction of range in phonological WM change scores. While phonological WM did not improve at the group level, there was considerable variability in the magnitude of performance change in phonological WM, with some individuals showing improvements and others showing decrements. In fact, the degree of variability in WM change was slightly higher for phonological (*SD* = 1.29) than for semantic WM (*SD* = 1.26). Thus, although there are many potential reasons for non-statistically significant effects, we consider a lack of variability in the change of phonological WM over time not a likely candidate.

The white matter tracts for which a significant correlation between FA values and semantic WM change scores was obtained were the left DAF, IFOF, and ILF. In the Horne et al. (2023, 2024) studies, which examined relations between white matter tracts and WM at a chronic time point when examining pairwise correlations between FA values and semantic WM, we obtained significant correlations for all three of these tracts. However, in the multiple regression results, a significant independent contribution of semantic WM to the prediction of FA values was obtained only for the IFOF. In fact, for the ILF, a significant independent contribution was obtained for phonological WM. For the DAF, neither semantic nor phonological WM had an independent contribution. As discussed in the Introduction, however, the predictions regarding the relation between tracts supporting WM at a given time point and those supporting recovery are not entirely straightforward. It is possible, for instance, that support from a tract supporting phonological WM (e.g., ILF) was helpful in providing a scaffold for the recovery of semantic WM, particularly if damage to tracts was severe—that is, providing the ability to maintain phonological representations that connect to semantic representations while mechanisms for semantic retention recover. For the DAF, it is possible that the tract supports aspects of WM common to both phonological and semantic WM (e.g., executive processes; Ribeiro et al., 2024) and the intercorrelation between the two WM measures prevented either type of WM from showing an independent contribution. Also, as noted earlier, it is possible that relations of semantic and phonological WM to tract integrity were different for the sample tested in Horne et al., which included many individuals with much larger lesions than those reported here (see Zahn et al., 2022). Only further studies having sufficient numbers of individuals at varying levels of tract disruption or lesion volume would be able to provide evidence regarding the potential contribution of these factors to tract involvement in recovery.

Another factor that may influence tract involvement during recovery is the presence or absence of language therapy. In prior studies of the role of white matter tracts in recovery, some have examined recovery without respect to the type or duration of therapy that might have been received (e.g., Keser, Meier, et al., 2020; Keser, Sebastian, et al., 2020; Schevenels et al., 2022), whereas other studies have examined the relation between behavioral treatment gains and the integrity of tracts at the outset of treatment or the relations between behavior changes and tract changes (e.g., Breier et al., 2011; Schlaug et al., 2009; Sihvonen et al., 2023). The former type often found language recovery was related to the left white matter tracts (Keser, Meier, et al., 2020). The few effects of right white matter tracts were negatively related to recovery (Keser, Sebastian, et al., 2020; cf. Sihvonen et al., 2023). In contrast, the latter type often revealed elevated white matter changes in the right hemisphere after therapies and such changes were related to degree of behavioral recovery (Schlaug et al., 2009; Wan et al., 2014). The current study was of the former type, examining WM change without respect to treatment. Although some individuals tested here may have received treatment for language disorders between the acute and chronic testing time points, it is highly unlikely that this treatment was directed at WM processes. Although arguably testing and treatment for WM deficits should be incorporated into standard speech/language therapy (e.g., Greenspan et al., 2024), such is not common practice.

Moreover, most studies on WM treatment have focused on phonological WM rather than both semantic and phonological WM (though see Harris et al., 2014). It is certainly possible that, if WM treatment was employed, greater involvement of right hemisphere tracts might be observed than was found here.

It should be noted, however, that a consensus seems to be emerging that there is no large-scale reorganization of language processes to the right hemisphere following brain damage (Wilson et al., 2023). Furthermore, others have argued that the terminology of “reorganization” is misleading, in that neural changes do not reflect a region taking on different functions than it did prior to brain damage (Makin & Krakauer, 2023). Instead, regions that become more involved during recovery are those that played some subsidiary role in that cognitive process prior to damage and the loss of regions more typically involved lead to greater activation in these subsidiary regions. Thus, to the extent that greater right hemisphere involvement is observed in language processes, such may reflect the enhancement of weak right hemisphere processing that persists in healthy individuals from childhood to adulthood (Martin et al., 2022). Interestingly, Makin and Krakauer suggest that greater changes in processing may occur in higher level regions that carry out more domain-general processes. On those grounds, one might have expected that WM processes would show greater right hemisphere involvement in recovery. However, as emphasized in the Introduction, the type of WM that was investigated here focused on the maintenance of language representations, rather than on complex executive processes which might lead to more right hemisphere involvement.

### Limitations

Our claims regarding the absence of right hemisphere tract involvement in WM recovery are based on null results. Some of the partial correlations for right hemisphere tracts had *p* values < 0.05, but were non-significant when controlling for multiple comparisons. Thus, it is certainly necessary to examine whether these null results would replicate in subsequent studies. Another concern is the absence of behavioral improvement in phonological WM at the group level. It is unclear why this occurred when semantic WM improved. As noted in the Results, three individuals showed a large decline in phonological WM, but excluding them did not result in a stronger positive relation of phonological WM and fiber tract integrity. One possible explanation for the lack of improvement in phonological WM is a difference in difficulty between tasks.

However, several points argue against such a possibility. Although mean performance was higher for the phonological WM task than the semantic WM task and the minimum span size for controls was higher for digit matching (5.0) than category probe (3.4), this is in part due to task design. Testing can start at a 1-item list length for category probe, whereas a 2-item list length is needed for digit matching in order to have an order reversal on non-matching trials. Testing controls and patients on increasing list lengths until their performance drops below 75% ensures that the difficulty of the two tasks is closely matched. Standard deviations of mean scores and of change scores were also similar for the two tasks. Thus, it does not appear that a ceiling effect prevented finding improvement on the digit matching task. Again, however, it would be valuable for future work to determine if our null findings for phonological WM can be replicated.

Another limitation was that only single measures of phonological WM and semantic WM were used in the current study, raising the issue of whether some other factor might differentiate performance on the two tasks, aside from the difference in the maintenance of phonological and semantic WM. In particular, the phonological WM task (digit matching span) had an order component whereas the semantic WM task (category probe) did not, and evidence suggests that there may be separable item and order components to WM (Majerus, 2019; Tian et al., 2022). Time limitations for testing individuals at the acute stroke stage prevented the administration of a large WM battery. We would note, however, that the digit matching task also required the retention of item information. If, for instance, a participant could retain only two items in a phonological form, then they should have difficulty on digit matching for lists with more than two items. That is, it is necessary to retain item information to encode item order. Prior results for individuals with left hemisphere stroke have shown a substantial correlation between digit matching span and a phonological WM task not requiring order retention (i.e., rhyme probe, in which participants judge whether a probe word rhymes with any item in a preceding list; *r*(36) = 0.58, *p* < 0.001; Zahn & Martin, 2024). Thus, it seems likely that there is a substantial contribution of item retention to individuals’ performance on digit matching span. Nonetheless, we cannot rule out the possibility that variation in the recovery of order retention ability was a major contributor to performance on digit matching span, leading to the different and non-significant relations to the white matter tracts that we obtained for digit matching.

### Conclusion

Here, we examined the degree to which white matter tracts in both left and right hemispheres contributed to the longitudinal recovery of two types of verbal WM, phonological and semantic WM from the acute to chronic stages of left hemisphere stroke. Tract integrity of the direct segment of the AF, the IFOF, and the ILF within the left hemisphere, but not the right hemisphere contributed to the recovery of semantic WM. We found no significant effects associated with phonological WM recovery in either hemisphere. To our knowledge, this is the first study relating bilateral changes in white matter tracts to the recovery of verbal WM after acute stroke. Our study provides several additional advances over previous work. Because we assessed WM performance within a few days after an initial left hemisphere stroke, we were able to assess the relationship between white-matter structure and function from before to after reorganization allowing a wider window for mapping the trajectory of recovery. Further, we examined this relationship within the same individuals, thus controlling for individual differences that are potential confounds in cross-sectional comparisons and, critically, allowing for a more accurate assessment of causality when examining the relationship between white-matter integrity and verbal WM. Overall, these results demonstrate that during the initial stages of recovery during the first year after stroke, the left hemisphere plays a significant role in recovery of verbal WM, thus providing support for the hypothesis that spontaneous recovery of language-related processes depends on connections that are pre-morbidly associated with function.

## ACKNOWLEDGMENTS

We gratefully acknowledge and thank our research subjects and their caregivers for their willingness to participate in this research. We thank Jolie Anderson, Miranda Brenneman, Cris Hamilton, Danielle Rossi, and Chia-Ming Lei for data collection. We thank Cris Hamilton for providing lesion mask demarcation. We thank the clinical neurological intensive care unit teams at the University of Texas Health Sciences Center and Memorial Hermann Hospital, the Houston Methodist Hospital, and the Baylor St. Luke’s Hospital for their assistance in patient recruitment and neurological assessment.

## FUNDING INFORMATION

Tatiana Schnur, National Institute on Deafness and Other Communication Disorders (https://dx.doi.org/10.13039/100000055), Award ID: R01DC014976. Randi Martin, T. L. L. Temple Foundation (https://dx.doi.org/10.13039/100020162).

## AUTHOR CONTRIBUTIONS

**Randi Martin**: Conceptualization: Lead; Funding acquisition: Supporting; Methodology: Equal; Writing – original draft: Lead. **Junhua Ding**: Formal analysis: Lead; Methodology: Equal; Visualization: Lead; Writing – original draft: Supporting. **Ali Alwani**: Formal analysis: Supporting. **Steve Fung**: Formal analysis: Supporting. **Tatiana Schnur**: Conceptualization: Equal; Funding acquisition: Lead; Methodology: Equal; Project administration: Lead; Supervision: Lead; Writing – original draft: Equal.

## DATA AND CODE AVAILABILITY

The analysis code, processed diffusion data, and behavioral data are available on OSF (Ding et al., 2025; https://osf.io/twe87/). T1 and diffusion data will be available upon reasonable request. Due to privacy concerns, T1 and diffusion data are currently not publicly available.
